# Prenatal diagnosis, genetic analysis, and pregnancy outcomes of fetuses with mosaic isodicentric Y chromosomes

**DOI:** 10.1186/s13039-026-00766-3

**Published:** 2026-04-27

**Authors:** Tieli Gao, Hua Wei, Yawen Zheng, Ying Xiao, Moling Zheng, Shurong Hong

**Affiliations:** Department of Molecular Genetic Center, Zhangzhou Affiliated Hospital of Fujian Medical University, Zhangzhou, China

**Keywords:** Idic(Y), Mosaicism, Chromosomal microarray analysis, Fluorescence in situ hybridization, AZF region

## Abstract

**Background:**

Isodicentric Y chromosomes [idic(Y)] are frequently detected in patients with disorders of sex development (DSDs). However, prenatal cases are rarely reported. We performed comprehensive prenatal diagnostic evaluations in three fetuses with mosaic idic(Y) and followed up on pregnancy outcomes to explore the genotype-phenotype correlation.

**Methods:**

Amniotic fluid cells from three fetuses were subjected to karyotyping and chromosomal microarray analysis (CMA), with confirmation by fluorescence in situ hybridization (FISH) and real-time quantitative PCR (qPCR). Subsequently, parental karyotypes were analyzed using peripheral blood samples to assess inheritance (de novo vs. inherited).

**Results:**

All three fetuses exhibited mosaic 45,X with idic(Y) karyotypes, which exhibited distinct breakpoints (Yp11.32, Yq11.221, and Yq11.223). Two fetuses had additional 46,XY cell lines. The SRY gene was retained in all cases. In Fetus 1, the AZF region was intact, whereas in Fetus 2 and Fetus 3, AZFb + c and AZFc were deleted. Fetus 1 was born alive following genetic counseling. Notably, a right adrenal neuroblastoma was detected during late gestation and successfully resected postnatally. The other two pregnant women elected for pregnancy termination.

**Conclusion:**

The integration of multiple technologies is essential for accurate prenatal diagnosis in cases of mosaic idic(Y). Our findings, including the rare association with neuroblastoma, contribute new insights into the phenotypic spectrum of this condition. Long-term follow-up and multidisciplinary clinical management are crucial for optimizing outcomes in live-born patients.

## Background

Disorders of sex development (DSDs) comprise a heterogeneous group of genetic conditions characterized by incongruence among chromosomal karyotypes, gonadal phenotypes, and anatomical sexual structures [1]. Fetal DSDs present significant challenges for birth defect surveillance and prevention [2].

Isodicentric Y chromosomes [idic(Y)] were first reported by Jacobs et al. [3] and represent a common clinical entity in DSD [4]. These structural abnormalities primarily arise through chromosomal recombination or sister chromatid fusion following Y chromosome breakage [5]. The dicentric nature of idic(Y) makes it unstable, frequently leading to mosaic patterns. This mosaicism involves multiple cell lineages, with 45,X being the most common [6]. Variability in breakpoints and mosaic proportions results from diverse Y chromosome breakage-fusion patterns [7].

The clinical presentation of patients with idic(Y) is highly variable. External genitalia may be male, female, or ambiguous. Manifestations range from classic Turner syndrome features, mixed gonadal dysgenesis, and short stature to isolated male infertility with azoospermia [8]. A subset of patients also experience neurodevelopmental comorbidities, including autism spectrum disorder and intellectual disability [9]. However, most DSD-related phenotypes emerge after puberty. In many cases, prenatal detection of idic(Y) is often prompted by noninvasive prenatal testing (NIPT) results or ultrasound abnormalities not associated with DSD. Owing to limitations of traditional prenatal screening methods and phenotypic latency, fewer prenatal than postnatal cases have been reported [10]. Therefore, more prenatal cases need to be studied to provide evidence for the diagnosis and long-term surveillance of idic(Y) patients. Notably, more molecular technologies have improved prenatal identification.

Here, we applied karyotyping, chromosomal microarray analysis (CMA), fluorescence in situ hybridization (FISH), and real-time quantitative PCR (qPCR) to analyze three cases of mosaic idic(Y), emphasizing the importance of combining multiple methods. After genetic counseling, we monitored pregnancy outcomes. Furthermore, we reviewed the literature to better understand the genotype-phenotype correlations.

## Subjects

Three cases who underwent invasive prenatal diagnosis at the Molecular Genetic Center of our hospital between January 2022 and December 2024 were included in our study. Indications for Case 1 and Case 2 were abnormal NIPT results, which reflected sex chromosome deficiency. The indication of Case 3 was ultrasound abnormalities, highlighting echogenic intracardiac focus and concomitant aortic narrowing. Amniotic fluid samples were collected from the three pregnant women, and peripheral blood was obtained from them and their spouses. General information about the pregnant women at prenatal diagnosis is listed in Table 1.

**Table 1:** General information about the three pregnant women at prenatal diagnosis

Case	Maternal age (y)	Gestational age (w)	Pregnancy history	Indication	Specimen
1	28	25	G1P0	NIPT abnormality	AF
2	28	18	G1P0	NIPT abnormality	AF
3	23	22	G2P1	Ultrasound abnormalities	AF

AF, amniotic fluid; y, year; w, week

## Methods

### Karyotype analysis

Amniotic fluid cells were centrifuged and cultured in Amniotic Fluid Cells Medium (HE NENG BIO, Guangzhou, China) for 8–10 days. Peripheral blood samples (~ 0.5 mL each) from the parents were cultured in Serum-free Lymphocytes Medium (HE NENG BIO) for 3 days. Cultured cells were harvested. Following colcemid treatment, hypotonic treatment, fixation, slide preparation, and G-banding, metaphase chromosomes were scanned for enumeration and structural analysis. The analysis was in accordance with the International System for Human Cytogenomics Nomenclature 2020 (ISCN2020).

### CMA

DNA was extracted from amniotic fluid samples of three pregnant women. CMA experiments were performed using Affymetrix CytoScan 750 K gene chip (Affymetrix, CA, USA) following the manufacturer’s instructions. The data were analyzed using Chromosome Analysis Suite software (Affymetrix, CA, USA).

### FISH analysis

For Case 1, interphase FISH was performed using a SRY/DXZ1 probe. For Case 2, interphase FISH was performed using a D18Z1/DXZ1/DYZ3 probe. For Case 3, metaphase FISH was performed using a SRY/DXZ1 probe. The SRY probe targets the sex-determining region (Yp11.3), while D18Z1, DXZ1, and DYZ1 hybridize to centromeric regions of chromosomes 18, X, and Y, respectively. Detection was performed following the manufacturer’s instructions using an Olympus BX53 fluorescence microscope with a Leica CytoVision analysis system.

### Analysis of sequence-tagged sites (STSs)

Y Chromosome Microdeletions Detection Kits (Tellgen Corporation, Shanghai, China) were used to detect deletions in the AZF regions of the Y chromosome. Each AZF region was assessed using two STSs: AZFa (sY84, sY86), AZFb (sY127, sY134), and AZFc (sY255, sY254). The internal controls included SRY for sex determination and ZFX/ZFY for gene integrity. These STSs were analyzed in two separate tubes using real-time fluorescence PCR to determine the presence of the corresponding target genes.

### Follow-up

Follow-up clinical data, including pregnancy outcomes, fetal sex, neonatal status at birth, and postnatal growth and development, were collected for the three cases.

## Results

Parental karyotypes were normal in all three cases, confirming that the chromosomal abnormalities in all three fetuses were de novo. The detailed detection results of the three fetuses are presented in Table 2.

**Fig. 1:**
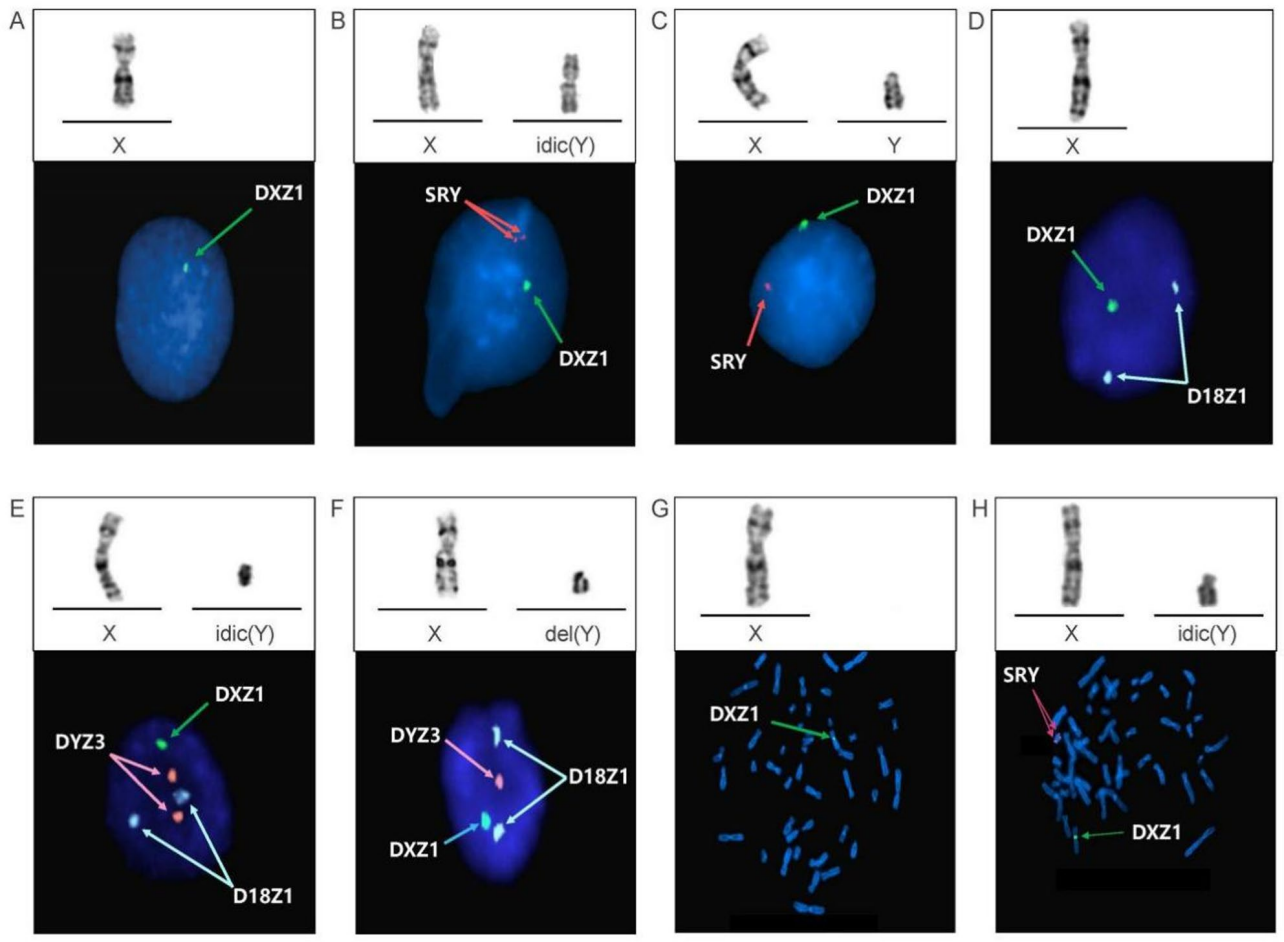
Karyotypes of the X and Y chromosomes for the three fetuses and FISH signals corresponding to the cell lines. Fetus 1: **A** 45,X. **B** 46,X,idic(Y)(p11.3). **C** 46,XY. Fetus 2: **D** 45,X. **E** 46,X,idic(Y)(q11.2). **F** 46,X,del(Y)(q11.2q11.2). Fetus 3: **G** 45,X. **H** 46,X,idic(Y)(q11.2)

**Fig. 2:**
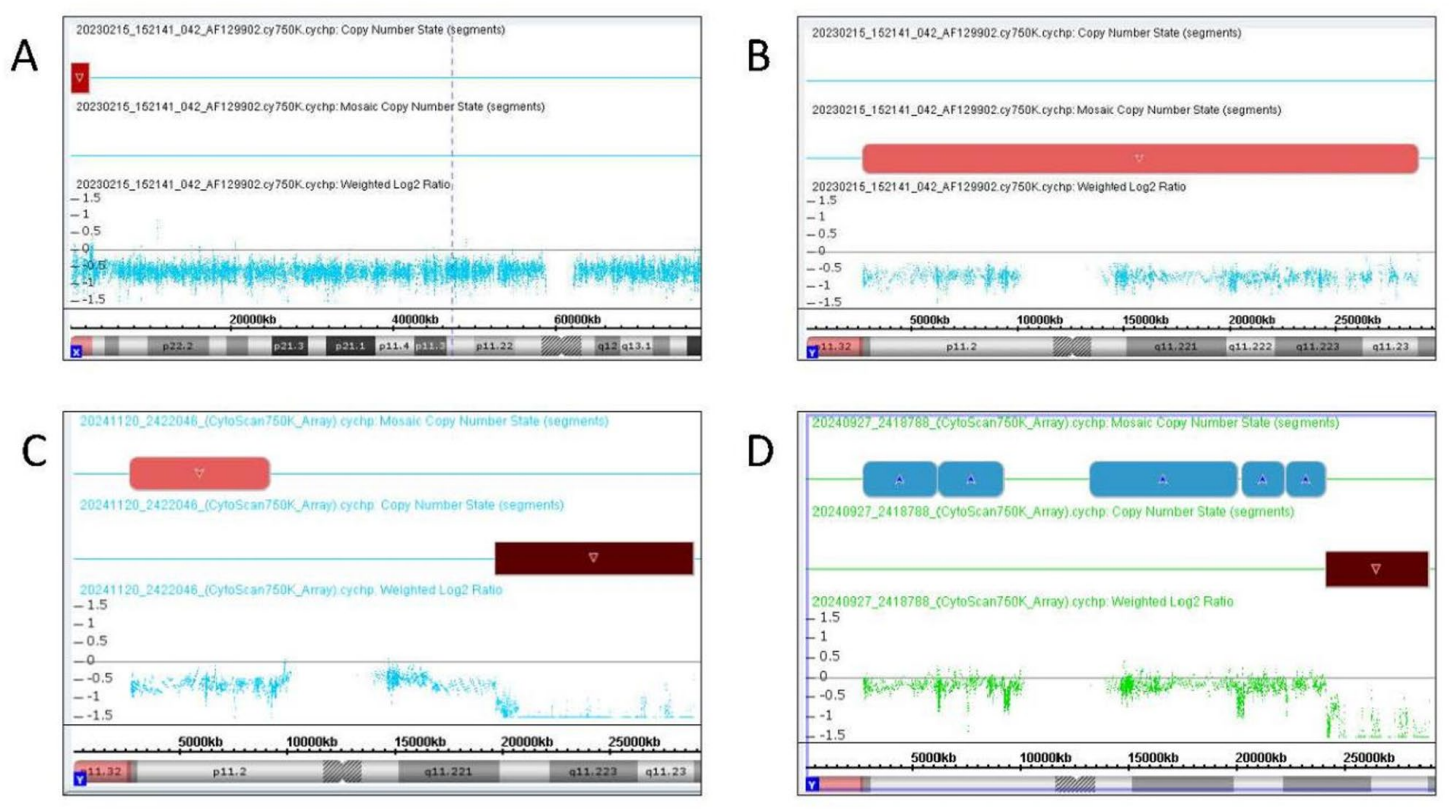
Chromosomal microarray analysis results for the three fetuses. The red part of (**A**) indicates the loss of one pseudoautosomal region of Fetus 1; the pink part of (**B**) indicates a mosaic deletion in the Y chromosome of Fetus 1; the pink and red parts of (**C**) indicate a mosaic deletion and a complete deletion in the Y chromosome of Fetus 2; and the blue and red parts of (**D**) indicate a mosaic duplication and a complete deletion in the Y chromosome of Fetus 3

**Fig. 3:**
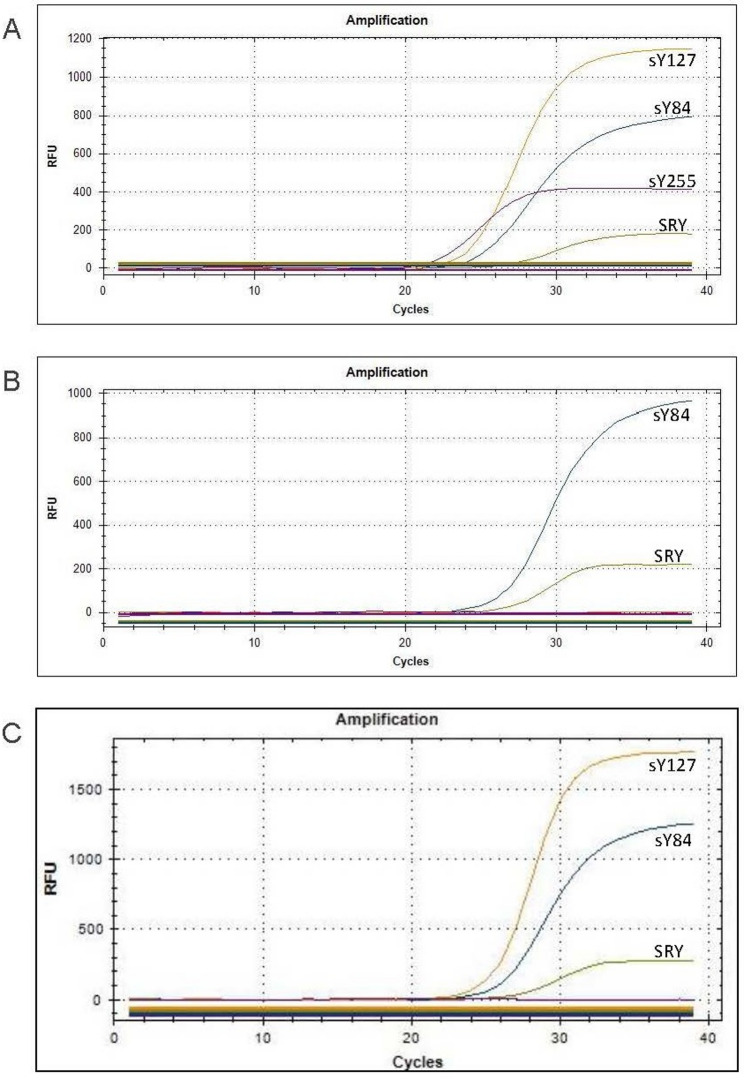
Y chromosome microdeletion detection results for the three fetuses. The amplification curves of the STSs revealed the following: Fetus 1: no microdeletion (**A**); Fetus 2: AZFb+c deletions (**B**); Fetus 3: AZFc deletion (**C**)

**Fig. 4:**
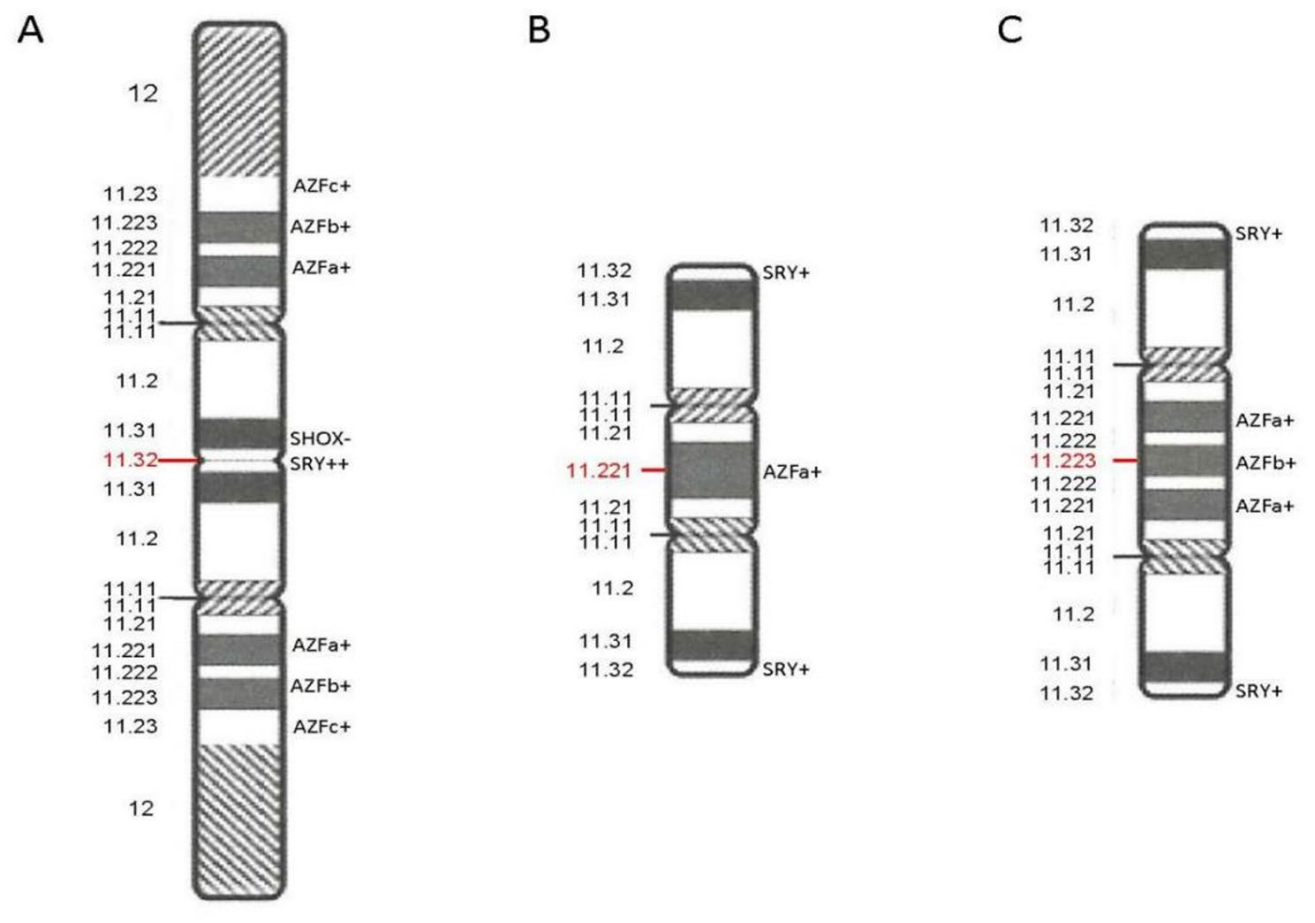
Schematic representation of idic(Y) structures in Fetus 1(**A**), Fetus 2(**B**) and Fetus 3(**C**)

The karyotype of Fetus 1 (fetus of Case 1) was 45,X[79]/46,X,idic(Y)(p11.3)[17]/46,XY[4] (Fig. 1A-C). Interphase FISH analysis using SRY/DXZ1 probes confirmed the same three cell lines (Fig. 1A-C) but with discordant mosaic proportions (Table 2) due to sampling bias. CMA revealed a 2.1 Mb deletion in the pseudoautosomal region. This region is located in Xp22.33 or Yp11.32 (Fig. 2A). CMA also revealed a 26.1 Mb mosaic deletion on the Yp11.31q11.23 segment (Fig. 2B). Based on these results and the results of the karyotyping, we concluded that the deleted pseudoautosomal region originated from Yp11.32. Deletion of this fragment resulted in SHOX gene haploinsufficiency, which may lead to short stature. Integration of CMA and karyotyping data identified Yp11.32 as the breakpoint (Fig. 4A). Although it was located near the SRY gene, the results of qPCR confirmed the presence of the SRY gene and all AZF regions (Fig. 3A).

**Table 2:** Detailed detection results for the three fetuses

Fetus	1	2	3
Karyotype	45,X[79]/ 46,X,idic(Y)(p11.3)[17]/ 46,XY[4]	45,X[85]/ 46,X,idic(Y)(q11.2)[10]/ 46,X,del(Y)(q11.2q11.2)[5]	45,X[65]/ 46,X,idic(Y)(q11.2)[35]
Breakpoint Location	Yp11.32	Yq11.221	Yq11.223
CMA Findings	arr[GRCh37] Xp22.33 or Yp11.32 (168552_2303322 or 118552_2253322)x1 Yp11.31q11.23(2650425_28799654)x0-1	arr[GRCh37] Yp11.31p11.2(2650425_ 9138568)x0[0.3] Yq11.221(16611169_ 19585046)x0[0.3] Yq11.221q11.23(19585047_ 28799654)x0	arr[GRCh37] (X)x1 Yp11.31q11.223(2650425_ 24073795)x2[0.4] Yq11.223q11.23(24073796_ 28799654)x0
FISH	nuc ish (DXZ1)×1,(SRY)×0[67]/(DXZ1)×1,(SRY)×2[15]/ (DXZ1,SRY)×1 [18]	nuc ish (DXZ1 × 1,DYZ3 × 1,D18Z1 × 2)[45]/(DXZ1 × 1,DYZ3 × 0,D18Z1 × 2)[44]/ (DXZ1 × 1,DYZ3 × 2,D18Z1 × 2) [11]	ish X(DXZ1)×1,(SRY)×0[33]/ X(DXZ1)×1,der(Y)(SRY++)[17]
qPCR	SRY(+) AZFa+b+c(+)	SRY+ AZFa(+) AZFb+c(-)	SRY+ AZFa+b(+) AZFc(-)
Ultrasound findings	Shorter fetal femur length, a right adrenal mass	Growth restriction	Aortic narrowing and hyperechoic bowel
Outcome	Birth, male, 2650 g at birth, born with right adrenal gland neuroblastoma, normal development at 23 months of age	TOP, male	TOP, male

TOP, termination of pregnancy

The breakpoints of idic(Y) in both Fetus 2 (the fetus of Case 2) and Fetus 3 (the fetus of Case 3) were located in Yq11.2. The karyotype of Fetus 2 was 45,X[85]/46,X,idic(Y)(q11.2)[10]/46,X,del(Y)(q11.2q11.2)[5] (Fig. 1D-F). The karyotype of Fetus 3 was 45,X[65]/46,X,idic(Y)(q11.2)[35] (Fig. 1G-H). Interphase FISH analysis of Fetus 2 revealed three mosaic cell lines (Fig. 1D-F). Metaphase FISH of Fetus 3 revealed two mosaic cell lines (Fig. 1G-H). According to the CMA results of Fetus 2, chromosome Y had a 6.5 Mb mosaic deletion on Yp11.31p11.2, a 2.9 Mb mosaic deletion on Yq11.221, and a 9.2 Mb complete deletion on Yq11.221q11.23 (Fig. 2C). CMA results for Fetus 3 revealed a 16.9 Mb mosaic duplication on Yp11.31q11.223 and a complete 4.7 Mb deletion on Yq11.223q11.23 (Fig. 2D). These results demonstrated that the precise breakpoints of idic(Y) in Fetus 2 and Fetus 3 were Yq11.221 (Fig. 4B) and Yq11.223 (Fig. 4C), respectively. qPCR results confirmed that Fetus 2 lacked AZFb+c regions (Fig. 3B), whereas Fetus 3 lacked only the AZFc region (Fig. 3C). A lack of these AZF regions may increase the risk of infertility in adulthood.

Following multidisciplinary consultation, the parents of Fetus 1 acknowledged the risk of poor fetal prognosis but opted to continue the pregnancy. For Fetus 2, ultrasound findings indicated growth restriction concurrently with karyotypic abnormalities, and the couple opted for pregnancy termination at 21 weeks of gestation. The pregnant woman in Case 3 underwent pregnancy termination at 26 gestational weeks. Following termination, both fetuses were phenotypically normal males.

Notably, ultrasound monitoring of Fetus 1 revealed a right adrenal mass at 37 weeks of gestation. The infant exhibited normal external genitalia at birth. At 1 month of age, surgical resection of the right adrenal mass was performed, with subsequent histopathological confirmation of neuroblastoma. A follow-up of this 23-month-old infant revealed a normal physical appearance and age-appropriate growth and development.

## Discussion

### Combined application of multiple technologies

Sex chromosome variations include diverse numerical and structural aberrations [11]. Precise genetic diagnosis using multiple technologies is the foundation for genetic counseling and pregnancy management.

The screening performance of NIPT for sex chromosome deficiency is lower than that for trisomy 21 because of the loss of the X chromosome with increasing maternal age [12]. Reduced total sex chromosome dosage may indicate abnormalities beyond monosomy X [13]. However, the NIPT results cannot identify the underlying complexity, such as the presence of a mosaic idic(Y) in Case 1 and Case 2. Further cytogenetic diagnosis to identify the detailed sex chromosome status is critical. Our cases highlight the role of NIPT as a screening tool to indicate idic(Y) and the importance of further diagnosis.

In prenatal diagnostic practice, karyotyping provides direct visualization of chromosomes, with resolution limited to ~ 5–10 Mb. Precise breakpoints were defined by integrating the CMA results. CMA revealed detailed chromosome copy number variations (CNVs) in three fetuses and breakpoint mapping. The specific hybridization of the probes renders FISH sensitive for detecting low-level mosaicism and allows direct enumeration of hybridized chromosomes and genes. In both Fetus 1 and Fetus 2, the proportions of 46,XY and 46,del(Y) were minimal, and the idic(Y) and del(Y) in Fetus 2 appeared similar. They were distinguished through FISH-based enumeration of Y chromosome probes (SRY or DYZ3). Moreover, the differences in mosaic percentage between the results of the karyotyping and FISH analyses may have been caused by the bias in cell culture and cell counting. The mosaic level identified by FISH is more reliable due to the uncultured cell state.

The breakpoints identified in our cases (Yp11.3 and Yq11.2) are recurrent [7], which may lead to deletions of the SRY gene or AZF regions. qPCR analysis yielded concordant results with the FISH and CMA findings, indicating the presence of the SRY gene and deletions in the AZF regions. In summary, the complementary application of multiple technologies provides comprehensive insights into these cases.

Formation mechanism and stability of idic(Y).

Current evidence suggests that paternal meiotic errors during gametogenesis represent the predominant etiology for mosaic idic(Y) formation [14]. The Y chromosome contains multiple palindromes [15], inverted repeats [16, 17], and AT-rich sequences [18, 19]. Sister chromatids of the Y chromosome are prone to breakage near these sites during separation. Subsequent crossover and recombination lead to fusion at breakpoints, resulting in the formation of idic(Y) while losing the acentric fragment [16]. The presence of two centromeres render idic(Y) unstable, leading to mosaic cell lines. Following fertilization by sperm carrying idic(Y), loss of the aberrant Y chromosome frequently occurs during early embryonic mitotic division, giving rise to 45,X cell lines and resulting in mosaic karyotypes.

Dynamic mosaicism could make the karyotypes even more variable [20]. The karyotype of amniotic fluid cells does not reflect that of neonatal peripheral blood [21]. Proportions of mosaicism can vary across tissues [17]. All three fetuses in our study exhibited high proportions of 45,X cell lines (65%~85%) but demonstrated normal male phenotypes at birth or termination. This may be attributed to the progressive decrease in 45,X cell lines during fetal development.

### Gene dosage effect

The 45,X cell line is the most common form of mosaicism with idic(Y), resulting in symptoms of Turner syndrome [7]. The SRY gene, located at Yp11.32, plays a critical role in male sexual differentiation [22]. Although Yp11.32 is a frequent breakpoint, most mosaic idic(Y) cases retain this gene [23, 24]. The presence of one or even two SRY copies does not guarantee a male phenotype, as the presence of 45,X cell lines may disrupt normal sex development [25]. The gene content of deleted Y-chromosome segments seems more important. As in Fetus 1 of our study, the deleted segment involved the short stature homeobox (SHOX) gene, which is a crucial regulator of chondrogenesis. SHOX gene haploinsufficiency may increase growth retardation in affected patients [26]. Therefore, both the 45,X cell line and SHOX gene haploinsufficiency may contribute to the short stature of Fetus 1.

When the breakpoint is located at Yq11.2, as in Fetus 2 and Fetus 3, the deleted segment may encompass the AZF regions, which contain the genes controlling spermatogenesis [27]. The deletions of different regions of AZFa, AZFb, and AZFc result in varying degrees of oligozoospermia or azoospermia [28]. The AZFb+c deletions manifest clinically as azoospermia [29, 30]. The AZFc deletions present highly variable phenotypes, ranging from normal semen parameters to severe oligozoospermia and even azoospermia [31]. Therefore, even if Fetus 2 and Fetus 3 were born as phenotypically normal males, they would still face risks of infertility in adulthood. Patients with AZFc deletions can be treated using intracytoplasmic sperm injection (ICSI), although this may transmit the deletion to male offspring. However, patients with AZFb+c deletions present with absolute azoospermia, making sperm donation the only viable path to parenthood[32].

### Correlation between the proportion of mosaicism and phenotype

No strong evidence supports a correlation between the postnatal sexual phenotype and the prevalence of mosaicism in prenatal specimens [33, 34]. The proportion of mosaicism in gonads seems to be more important. The impact of gonadal 45,X/idic(Y) mosaicism on gonadal development may be analogous to that of mixed gonadal dysgenesis caused by the 45,X/46,XY karyotype [35, 36]. High levels of the 45,X cell line in male gonads may lead to the haploinsufficiency of SRY, mosaic loss of the key genes, and impair the ability to maintain normal testicular differentiation, resulting in ambiguous genitalia [36]. In female patients with Turner syndrome-like phenotypes, Y chromosome material mosaicism in the gonad increases the risk of gonadoblastoma [37]. During the prenatal period, a key limitation is the inability to access potentially affected tissues, such as gonadal tissue. Future studies on mosaicism patterns in affected tissues of idic(Y)-bearing children may enhance the ability to predict phenotypes.

### Prenatal counseling and postnatal management

In most postnatal cases with mosaic idic(Y), karyotype abnormalities are typically detected due to the presence of abnormal phenotypes, biasing the available data[4, 38]. Although relatively rare, prenatal cases appear to have more favorable outcomes [39].

Yang et al. [34] reported six new idic(Y) cases, with a comprehensive review of 31 previously documented cases. By combining recent reports [21, 40, 41] and 3 additional cases from our study, we reviewed a total of 50 prenatal cases with idic(Y). Among the 50 fetuses, 39 were male, 3 were female, 3 had ambiguous gonads, and 6 were of undetermined sex. Thirty-two of the 50 fetuses were delivered, 15 pregnancies were terminated, and three cases had unknown outcomes. It can be concluded that most fetuses carrying idic(Y) were born as phenotypically normal males. Among the live-born fetuses, 8 presented with varying abnormalities—mostly ambiguous genitalia, short stature, or cardiac defects. Other rare abnormalities included complex heart lesions [42], multiple structural malformations [43], and right adrenal gland neuroblastoma in Fetus 1 of our study. These abnormalities cannot be explained by the idic(Y) karyotype and may be related to individual environmental/developmental factors or limited sample size.

Most symptoms associated with chromosome abnormalities typically manifest during or after puberty, with increased risks of infertility and gonadal dysgenesis in males or Turner syndrome manifestations and gonadoblastoma in females. Therefore, prenatal ultrasound evaluation of fetal external genitalia and regular postnatal follow-up until at least puberty are critically important [44]. Prenatal counseling should be conducted appropriately. Postnatal management requires risk identification and corresponding intervention recommendations. In our study, Fetus 1 underwent timely surgical resection of the adrenal tumor, with no recurrence observed and with normal growth development observed to date. The parents have demonstrated excellent compliance with follow-up surveillance, representing an ideal management model that we hope to replicate.

## Conclusion

Through the integrated application of multiple genetic technologies, we successfully diagnosed three fetuses with mosaic idic(Y), offering novel insights into the genotypic and phenotypic spectrum of this rare chromosomal abnormality. Our findings underscore the need for precise molecular characterization and a thorough understanding of the underlying mechanisms to inform effective prenatal genetic counseling. Furthermore, long-term postnatal follow-up and individualized management remain crucial for monitoring developmental outcomes and addressing potential complications, thereby ensuring optimal care for affected individuals.

## Data Availability

The data that support the findings of this study are available from the corresponding author upon reasonable request.

## Abbreviations

Idic(Y): Isodicentric Y chromosomes
DSD: Disorders of sex development
CMA: Chromosomal microarray analysis
FISH: Fluorescence in situ hybridization
qPCR: real-time quantitative PCR
AF: Amniotic fluid
ISCN2020: International System for Human Cytogenomics Nomenclature 2020
NIPT: Noninvasive prenatal testing
STSs: Sequence-tagged sites
SHOX: Short stature homeobox
